# Evaluations of digital public health interventions in the WHO Southeast Asia Region: a systematic literature review

**DOI:** 10.1017/S026646232400045X

**Published:** 2024-12-18

**Authors:** Nachiket Gudi, Elstin Anbu Raj, Beate Jahn, Uwe Siebert, Angela Brand

**Affiliations:** 1 Department of Health Information, Prasanna School of Public Health, Manipal Academy of Higher Education, Manipal, Karnataka, India; 2 Faculty of Health Medicine and Life Sciences (FHML), Maastricht University, Maastricht, The Netherlands; 3 Department of Pharmacy Practice, Manipal College of Pharmaceutical Sciences, Manipal Academy of Higher Education, Manipal, India; 4 Institute of Public Health, Medical Decision Making and Health Technology Assessment, Department of Public Health, Health Services Research and Health Technology Assessment, UMIT TIROL – University for Health Sciences and Technology, Hall in Tirol, Austria; 5 Division of Health Technology Assessment, ONCOTYROL – Center for Personalized Cancer Medicine, Innsbruck, Austria; 6 Center for Health Decision Science, Departments of Epidemiology and Health Policy & Management, Harvard T.H. Chan School of Public Health, Boston, MA, USA; 7 Program on Cardiovascular Research, Institute for Technology Assessment and Department of Radiology, Massachusetts General Hospital, Harvard Medical School, Boston, MA, USA; 8 United Nations University – Maastricht Economic and Social Research Institute on Innovation and Technology (UNU-MERIT), Maastricht, The Netherlands

**Keywords:** digital health, HTA, m-Health, systematic literature review, telemedicine, EUnetHTA

## Abstract

**Introduction:**

Digital health technologies have been enhancing the capacity of healthcare providers and, thereby, the delivery of targeted health services. The Southeast Asia Region (SEAR) has invested in strengthening digital public health. Many digital health interventions have been implemented in public health settings but are rarely assessed using the holistic health technology assessment (HTA) approach.

**Methods:**

A systematic literature review was performed to provide an overview of evaluations of digital public health interventions in the World Health Organization (WHO) SEAR. Searches were conducted on four electronic databases. Screening title abstracts and full texts was independently conducted by two reviewers, followed by data extraction. Dimensions of HTA were analyzed against the EUnetHTA Core Model 3.0. Quality assessment of included articles was conducted using the JBI Checklist for Economic Evaluation and Consolidated Health Economic Evaluation Reporting Standards 2022 checklist to assess the reporting quality. The findings are presented using systematic evidence tables and bar charts.

**Results:**

Of the forty-three studies screened at the full-text stage, thirteen studies conducted across six countries were included in the analysis. Telemedicine and m-health interventions were assessed in ten studies. Nine studies conducted cost-effectiveness analysis, and five assessments were conducted from a societal perspective. Four studies utilized more than one perspective for the assessment. Health problem definition and current use of technology, description and technical characteristics of the technology, clinical effectiveness, costs, economic evaluation, and organizational aspects were assessed by all the studies, whereas legal aspects were least assessed.

**Conclusion:**

The lack of HTAs on digital public health interventions in the region highlights the need for capacity-building efforts.

## Introduction

Digital health technologies (DHTs) have been at the forefront of healthcare reforms in the last decade and more so in public health settings. Although the Director General of the World Health Organization (WHO) reckoned that the future of healthcare is digital, the pandemic catalyzed that journey for the developing world (1). Various digital health innovations were implemented during the pandemic (2). DHTs include computing platforms, connectivity, software, sensors, and other interventions delivered through an interface (3). DHTs are implemented to improve healthcare, reduce costs, and increase equity, enhancing access to care (4). DHTs have often been considered disruptive in the healthcare domain (5). These technologies enter the market through two routes: first, through the clinical trial route, and, second, through the direct market route, where there is limited testing and evaluations for clinical efficacy. With the emergence of e-commerce platforms, it has become easy for these technologies to percolate the healthcare market, surpassing the traditional trial route. With the startup culture gaining momentum in developing economies, various products are directly entering the market with limited evidence to support their efficacy and safety claims.

Those DHTs that are entering the market through the clinical trials route mature from the stage of pre-prototype to implementation and scale-up (although not sequentially) (6). Investments are made not only on the clinical spectrum to render patient care but also on the public health side, where the emphasis is on disease prevention and health promotion for the masses or the community. Huge investments are made in DHTs, with development agencies and governments at the forefront by creating ecosystems that enable these technologies to thrive in the healthcare market (4,5,7,8). The WHO Southeast Asia Region (WHO SEAR) has been dynamic in rolling out digital technologies and creating national digital health strategies due to the aging population, shortage of human resources for the health sector, change in spending patterns on health, and environment fostering technological innovations (internet penetration, cost of one gigabyte of internet data, and mobile phone users) (8). India launched the Ayushman Bharat Digital Mission in September 2021 (9). Bangladesh has initiated preparing its national digital health strategy with the WHO SEARO Regional Office (10). Nepal launched its National e-Health Strategy in 2017 and has further emphasized the use of DHT in its country’s cooperation strategy with the WHO (11). The countries of the WHO SEAR have also shown resilience during COVID-19 through their telemedicine practice guidelines to support technology-aided healthcare delivery (12).

With considerable investments in creating technology infrastructure, various digital health interventions (DHIs) being deployed, and changing paradigms from clinical to clinical and cost-effectiveness to the current day patient-related, legal, and organizational dimensions of a health technology, there is a need to understand how these technologies deployed for the public health have been evaluated in the WHO SEAR. Although the phrase DHTs represents a plethora of interventions in general and is often used as an umbrella term, we operationalize the definition of digital public health intervention as “any health care intervention delivered using information and communication technology at the community level/population to achieve population-level health objectives, although the unit of analysis may be at the individual level (care delivered to a patient outside the hospital setting).” Evidence suggests that there are a few frameworks to assess DHIs (13,14).

Health technology assessment (HTA) in the SEAR has gained momentum, with the HTAsiaLink network being one of the important initiatives (15). Countries such as India, Thailand, and Bangladesh are investing in strengthening their digital public health infrastructure. With digital health gaining impetus among the low–middle-income countries (LMICs) and more so in the WHO SEAR, it becomes imperative to understand the assessments of digital public health interventions in the region. This facilitates identifying various digital health modalities evaluated, methodologies used, and opportunities for capacity building. We utilized a systematic literature review approach to provide an overview of the evaluations of digital public health interventions in the WHO SEAR. Understanding the assessment landscape can inform various key stakeholders in making these technologies safe, accessible, acceptable, and affordable to individuals.

## Methodology

## Search

Searches were conducted on the following four electronic databases: PubMed (NCBI), Scopus (Elsevier), Embase (Elsevier), and Web of Science (Clarivate). The search was restricted for the years 2010–2022, as we observed an increasing trend in published records from 2010 on PubMed (NCBI). Keywords used are public health, DHI, HTA, biomedical assessment, economic evaluation, and community. All the records were imported into Rayyan (16), and deduplication was conducted. A comprehensive list of search strategies is given in Supplementary Table S1. A protocol was established *a priori.* We did not publish the protocol.

## Study selection

The selection of the studies was conducted at two sequential stages: title abstract (Ti-Ab) and full text (FT), conducted independently by two reviewers (NG and EAR). A consensus-building approach was used to resolve conflicts on the selection of the article in the presence of an arbitrator (AB). The Preferred Reporting Items for Systematic Reviews and Meta-Analyses (PRISMA) 2020 chart (17) was used to document articles at each stage of the review based on the following selection criteria:

## Eligibility criteria

We included peer-reviewed literature of primary and secondary data studies conducted in the WHO SEAR, published in English only. A list of articles excluded at the full-text stage, along with the reasons for exclusion, is mentioned in Supplementary Table S2. The selection criteria are given in Table 1.

**Table 1.** Selection criteria

Inclusion criteria	Exclusion criteria
**Population:** Any population, irrespective of age, disease condition, and severity of disease, who benefits from a DHI in a community setting. **Intervention:** We included any digital public health intervention, including AI-based interventions. We followed the WHO Classification of DHI V1.0’s definition, “discrete functionality of digital technology that is applied to achieve health objectives,” to define DHI (18). **Comparator:** Any comparator, including studies without comparators, do-nothing scenarios, the standard of care, or alternative interventions, was eligible for inclusion. **Outcomes:** Because the objective of the study was to map and later understand the evaluation of the HTA dimensions of DHIs, we did not limit the inclusion based on the outcomes. We documented the reported study outcome. **Study setting:** We have included only studies conducted in a community-based setting or a population-based setting within the WHO SEAR (Bangladesh, Bhutan, DPRK, India, Indonesia, Maldives, Myanmar, Nepal, Sri Lanka, Thailand, and Timor-Leste) (19)	We have excluded articles not published in the English language. We excluded articles classified as reviews, editorials, letters, commentaries, perspectives, opinions, reports, and conference abstracts, as the intention was to map and systematically assess the HTA dimensions of DHI. We excluded articles related to vaccines, biologicals, pharmacological products, biomarkers, diagnostic studies, therapeutic trials, predictions, simulations, infection control, management of COVID–19, protocols, and preprints.

AI, artificial intelligence; DHI, digital health intervention; DPRK, Democratic People’s Republic of Korea; HTA, health technology assessment.

## Data extraction

Two individuals (NG and EAR) independently extracted and reconciled data to minimize inconsistencies. Data were extracted for study objective, study design, country of implementation, settings, disease targeted, type of intervention, type of economic evaluation, perspective of economic evaluation, observation period, and outcome measures. The dimensions from the core model of HTA are assessed as espoused by the EUnetHTA Core Model 3.0 (20). Thus, our data extraction sheet was flexible to accommodate additional information. The data extraction sheet was pretested on three included studies to ensure comprehensiveness. We did not make any author contact for missing information. While the summary of the study characteristics is presented as a table here, details, including results, limitations, and assessment of the core model of HTA, are given in the Supplementary Material.

## Assessment of conducting and reporting quality

The quality of the included interventions was assessed using the JBI Checklist for Economic Evaluation (21). The scores were converted to percentages and later classified into “well conducted,” “moderately well conducted,” and “poor quality.” The study team agreed upon this classification and the score intervals, as there was no standard guidance on the scoring system. The studies that secured a score greater than 90 percent were classified as “well conducted,” those between 80 percent and 90 percent were adjudged as “moderate,” and those that scored below 80 percent were classified as “poor-quality” studies. In the case of items that were not clear, a consensus-building approach was employed to arrive at a decision. The reporting quality of the included studies was assessed against the Consolidated Health Economic Evaluation Reporting Standards (CHEERS 2022) statement, as it could be used for any form of health economic evaluation (both primary and secondary data studies) (22). The quality assessment of the study’s conduct and reporting was carried out independently by two reviewers (EAR and NG). A clustered column chart is utilized to demonstrate the reporting quality of each item. The critical appraisal and completeness of reporting are given in a table in the Supplementary Material.

## Data synthesis and reporting

Results are summarized as frequencies and reported using tables and figures. The review is reported according to the PRISMA 2020 statement (17).

## Results

**Figure 1.**
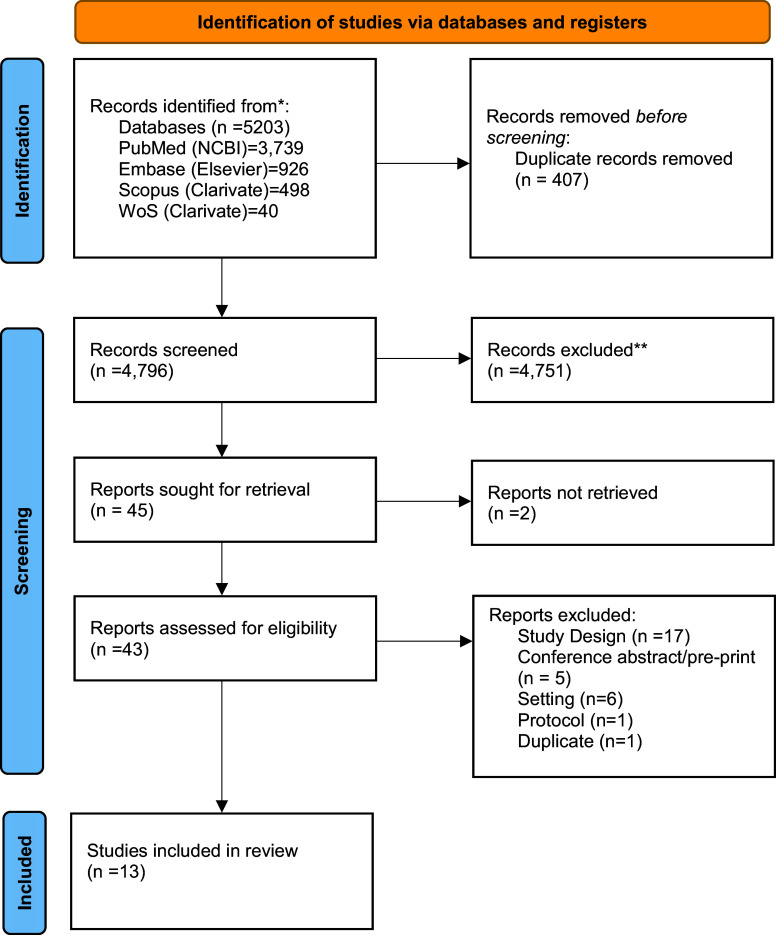
PRISMA flow diagram showing records at each stage of the review.

The search yielded a total of 5,203 articles from various databases. After the removal of 407 duplicates, 4,796 were subjected to screening at the Title–Abstract stage. Four thousand seven hundred and fifty-one articles were excluded, and forty-five of them were considered for full-text screening. We could not retrieve two articles. We excluded thirty articles and included thirteen articles for the analysis. A list of excluded articles at the full-text stage and the respective reasons for exclusion are given in Supplementary Table S2. The study characteristics of the included studies are given in Supplementary Tables S3 and S4. Articles at each stage of the review are pictorially represented using the PRISMA flow diagram (Figure 1) (17).

## Characteristics of the studies/study design and location

Studies included in the review were published between 2013 and 2021. The studies employed different designs such as multi-methods approach (Menon 2021 [India]) (23); quasi-experimental design (Angell 2021 [Indonesia] (24) Jo 2021 [Bangladesh] (25), Jo 2019 [Bangladesh] (26)); non-randomized open-labeled design (Wongwai 2015 [Thailand] (27)); randomized controlled design (Salvadori 2020 [Thailand] (28), Arora 2017 [India and Bangladesh] (29)), Cluster randomization approach (Anchala 2015 [India] (30), Modi 2020 [India] (31)); modeling approach (Xie 2020 [Singapore] (32), Rachapelle 2013 [India] (33)); retrospective-observational study (Thakar 2018 [India] (34)); and a hypothetical cohort design (Nguyen 2016 [Singapore] (35)).

## Disease/condition/procedure assessed and the DHI implemented

Of the thirteen studies, five assessed telemedicine (23,27,33–35), five assessed m-health interventions (24–26,28,31), one assessed decision support systems (30), and one of the studies assessed artificial intelligence (AI)-based intervention for teleophthalmology-based diabetic retinopathy screening (32) and one was a telephone-based supportive management for pressure ulcers (29).

The telemedicine interventions were intended to manage casualties among the armed forces at the Ladakh border in India (23), screening for retinopathy (32,33) diabetic retinopathy of prematurity (27), and consultations for patients who underwent elective neurosurgery (34). The m-health interventions were assessed for the management of cardiovascular risk (24), the provision of maternal and newborn health services (25,26), remainders for re-testing for HIV (28), and improving infant mortality rates in Gujarat (31). Decision support systems were tested to manage hypertension in primary healthcare settings (30). AI was not used as a standalone intervention but combined with a teleophthalmology-based diabetic screening intervention (32). A summary of the included studies is given in Table 2.

**Table 2.** Summary of included studies

Study design	Country of intervention implementation	Digital health modality	Disease/condition
Randomized controlled trial	Thailand (28)	m-health	HIV
	India and Bangladesh (29)	Telephone-based support	Management of pressure ulcers in people with spinal cord injury
	India (30)	DSS	Hypertension
	India (31)	m-health	Childcare
Quasi-experimental design	Indonesia (24)	m-health	CVD risk mgmt.
	Bangladesh (26)	m-health	Maternal and newborn services
	Bangladesh (25)	m-health	Maternal and newborn services
modelling	Singapore (32)	AI with Teleophthalmology	Diabetic retinopathy screening
	India (33)	Telemedicine	Diabetic retinopathy screening
Multi-methods	India (23)	Telemedicine	Casualty management by armed forces
Non-randomized open-labeled trial	Thailand (27)	Telemedicine	Retinopathy of prematurity
Retrospective observational study	India (34)	Telemedicine	Post-surgery follow-up
Hypothetical cohort design	Singapore (35)	Telemedicine	Diabetic retinopathy

AI, artificial intelligence; CVD, cardiovascular disease; DSS, decision support system; HIV, human immunodeficiency virus.

## Perspective/time horizon and outcomes of evaluations

Ten studies conducted cost-effectiveness analysis (23–26,28–31,34,35), two studies performed cost-utility analysis (27,33), and one study performed cost-minimization analysis (32). Six of the thirteen evaluations were conducted from a societal perspective (25,26,28–30,34), and three were conducted from a health systems perspective (23,24,32). Three evaluations were conducted from a healthcare provider and societal perspective (27,31,33). One evaluation was conducted from a societal and health systems perspective (35). The observation period for the evaluations ranged between 3 months (29) and a lifetime horizon (27,35).

Different outcome measures captured were segregated into health outcomes (clinical effectiveness, disability-adjusted life years (DALY), quality of life), and economic outcomes (incremental cost-effectiveness ratios [ICER], annual treatment costs, utility score, healthcare, and productivity cost).

## Assessment of dimensions from the core model of HTA

We further assessed the various domains under the EUnetHTA 3.0 core model of HTA as covered by these evaluations. All the assessments detailed on the “Health problem and Current Use of Technology (CUR),” “Description and technical characteristics of technology (TEC),” “Clinical effectiveness (EFF),” “Costs and economic evaluation (ECO),” and the “Organizational aspects (ORG).” Three studies assessed “the Safety (SAF)” of the intervention (23,31,33). Only two studies reported on the “Ethical Analysis (ETH)” (23,31). “Patient and Social Aspects” were reported in more than half of the evaluations (seven studies) (23,25,28,31–34). Legal aspects were assessed in one study (23). The assessment dimensions are given in Supplementary Table S5. A bar chart showing the dimensions from the EUnetHTA Core Model 3.0 covered by the included studies is given in Supplementary Figure S1.

## Critical appraisal of the economic evaluations

We assessed the quality of the included economic evaluations based on the “JBI Checklist for Economic Evaluations” (21). Nine of the thirteen studies were well conducted; two were moderately well conducted, whereas two were of poor quality. Supplementary Table S6 presents the results of the critical appraisal. A bar chart of the critical appraisal of the included studies is given in Supplementary Figure S2.

## Completeness of reporting

All the studies reported on items 4–9, 11–14, 16, 22, 26, and 28 of the CHEERS 2022 statement. If an item was marked as “not applicable,” we have not considered it for assessing the completeness of reporting. Reporting of each item in the CHEERS 2022 checklist is given in Supplementary Table S7. A clustered column chart is given in Supplementary Figure S3 to provide an overview of the reporting practices, where the x-axis represents the items in the CHEERS 2022 checklist, whereas the y-axis represents the completeness of reporting.

## Discussion

This systematic literature review found that there is a limited yet growing body of evidence for the evaluations of digital public health interventions in the WHO SEAR. Of the thirteen studies from six countries that were included and assessed, ten received funding (24–28,30–33,35). Four of the ten assessments that were funded, received grants from government agencies (27,31,32,35). Three of these four government-funded assessments were for the evaluation of telemedicine (27,31,35), and one was for the evaluation of AI along with telemedicine (teleophthalmology) (32). This could be due to various levels of political support, lack of systematic early dialogues, access to health information and technology, and the country’s HTA bodies/nodal agencies. Similar findings were observed by Sharma et al. (36) in their landscape analysis of HTA capacity among ASEAN countries, where they found that Malaysia, Singapore, and Thailand have well-developed HTA capacities, and other nations are making an effort to develop HTA capacity (36). No literature was found, particularly from the Democratic People’s Republic of Korea and Timor-Leste; the same was also observed by Goel et al. (37) in their scoping review.

Of the various DHIs mentioned in the WHO DHI Classification V1.0 (18), we found that majority of them were evaluations of telemedicine and m-health interventions, whereas there was only one assessment of an AI-based intervention. Three of the five telemedicine interventions were driven/supported by the government (23,27,35), whereas one of the five m-health assessments were funded by the government (31). This highlights the importance of telemedicine and its growing need among LMICs. Many countries in the WHO SEAR have been making strides in telemedicine deployment in healthcare through enabling policy and infrastructure support, although most of it was evident during the COVID-19 pandemic (12). The growing popularity as compared to other modalities may be attributed to its ease of operation, limited one-time cost of setting up the infrastructure, and governments’ support to its deployment as it minimizes the overall cost of seeking primary care (12). Although we used the DHI Classification V1.0’s (18) definition for DHI, we felt the need for a broader and harmonized definition for DHI.

The m-health interventions were not disease-specific and offered a bundle of services for managing cardiovascular risk, maternal and child health (MCH) services, and improving mortality rates. Similar observations were made by Godinho et al. (38) in the Western Pacific Region. Despite the growing debate on using AI in healthcare, only one study was found in the public health setting within the SEAR. This could be attributed to delayed adoption and evaluation of AI within the region. A limited number of clinical trials on AI in healthcare have been observed by Lam et al. (39) and Wang et al. (40), thus calling for efforts in capacity building and enhanced funding opportunities for their assessments.

We did not come across an evaluation of applications (apps) in our review. This could be due to the need for guidance in evaluating these dynamic applications. The time interval between the development and implementation of these apps is short and therefore challenging to assess (14). Another reason for limited assessments of DHI from an HTA perspective is attributed to fewer HTA frameworks tailored to evaluate the DHI, and this assertion is further echoed by Haverinen et al. (13) and Kolasa et al. (41).

All evaluations stated, “Health problem and Current Use of Technology (CUR),” “Description and technical characteristics of technology (TEC),” “Clinical effectiveness (EFF),” “Costs and economic evaluation (ECO),” and the “Organizational aspects (ORG).” Only two evaluations reported “Ethical Analysis (ETH)” and one study detailed about "Legal Aspects (LEG),” thus highlighting the need for considering existing moral and social norms about the technology being evaluated as well as the legal national and global aspects. To enhance equitable access to DHI, “Ethical analysis (ETH)” and “Patients and Social Aspects (SOC)” domains of the EUnetHTA 3.0 have to be incorporated while performing a holistic HTA (20). Although most of the outcomes used in economic evaluation, such as QALY and DALY, among others for health outcomes and ICERs for cost-effectiveness, were captured, no studies were found assessing usability outcomes, likelihood to recommend, feasibility, and other technology adoption outcomes. This may be the case because HTAs have been revolving around costs and health consequences, with limited importance being laid on how these consequences are enabled through the deployment of DHI. Recent developments in the digital health world include using Software as a Medical Device (42) and monetizing data for the public (health) good. These developments will call for understanding the public trust in the data captured and the benefits of monetizing that data (43).

Overall, the included studies have been well conducted and sufficiently well reported except for detailing the engagement of stakeholders in designing the analysis (Item 21) and “reporting on the difference made to the study when stakeholders were engaged” (Item 25). The involvement of stakeholders, including patients, in the design of HTA studies would enhance the uptake of evidence to inform policy and practice. It is further observed that while reporting on item 27 (“Describe how the study was funded and any role of the funder in the identification, design, conduct, and reporting of the analysis”), there was an insufficiency of reporting as most of the studies reported regarding the source of funding and refrained from detailing the role of the funder in the identification, design, conduct, and reporting of the analysis. The findings of this systematic literature review are in coherence with observations by Flemming et al. (44). “How to report and how much to report” has been a historical challenge to researchers, and under-reporting studies will raise questions about the validity of the study. Reporting quality is also affected by prescribed word counts for abstracts and full texts, and collective efforts by journal editors, peer reviewers, and authors to enhance reporting standards will be beneficial (45).

Thus, to summarize, HTA is emerging in the WHO SEAR, calling for stronger capacity-building needs; need for active stakeholder engagement through systematic early dialogues; need for a harmonized definition of DHIs, and stronger political will for the adoption of DHI and HTA within the WHO SEAR.

## Strengths and limitations of the study

As a strength, this systematic literature review has focused on the countries of WHO SEARO, where the HTA landscape is evolving. This analysis is expected to stimulate the current debate on three aspects: (i) Limited diversity in the modalities of DHIs evaluated from an HTA lens, (ii) evaluations focusing on clinical and economic lens with limited importance to ethical, social, and legal aspects, and (iii) conducting and reporting aspects of these interventions.

Our systematic literature review also has a few limitations. First, although DHIs for public health (or digital public health interventions) have been extensively piloted in this region, there is still limited literature available on the academic databases. This could be attributed to the low publication of HTA reports but accessible through a gray literature search (publication bias). Second, the new CHEERS checklist was published in 2022, which could have influenced the judgments made on the reporting quality. Although some studies mentioned that they had utilized the CHEERS checklist (an earlier version of the guideline), they had been subjected to the assessment according to the CHEERS 2022 checklist. The CHEERS (2022) make four additions to the CHEERS (2013) checklist. They are (i) “Health economics analysis plan,” (ii) “Characterizing distributional effects,” (iii) “Approach to engagement with patients and others affected by the study,” and (iv) “Effect of engagement with patients and others affected by the study.” Third, authors had not been contacted in case full texts to published protocols were unavailable and gray literature sources were not included in the systematic literature review. The protocol was not registered or published, although the protocol was established *a priori.* The dimensions from the EUnetHTA Core Model 3.0 have been used, although it specifically caters to the European context. Finally, Drummond’s checklist to critique the included studies was chosen first, but later the JBI Critical Appraisal tool was used for economic evaluation and reported as a deviation from the protocol as the latter caters to the appraisal of both primary and secondary data studies. Further efforts may synthesize evidence from the clinical settings. Employing a gray literature search using reports from the HTA agencies to locate the literature may offer a more comprehensive list of studies. Future systematic literature reviews may explore evaluations of DHI from an HTA perspective among other developing countries. There is a stronger need for capacity building in evaluating DHIs from an HTA lens. This research also highlights the need for a harmonized definition of DHI.

## Conclusion

The systematic literature review identified and included thirteen studies using HTA dimensions of digital public health interventions, which were well conducted and fairly well reported across six different countries in the WHO SEAR with an emphasis on telemedicine and m-health modalities. Although the HTA approach to implementing and reimbursing interventions has been initiated, such endeavors need to be strengthened. The limited set of HTAs of digital public health interventions highlights the need for additional evaluations, funding, and stronger collaborations between the government and other stakeholders, as digital public health interventions aim to ensure governmental uptake. A stronger need for capacity building and frameworks to assess DHI from an HTA lens is required.

## Supporting information

Gudi et al. supplementary material Gudi et al. supplementary material

## Supplementary material

The supplementary material for this article can be found at http://doi.org/10.1017/S026646232400045X.

## Credit statement

Conceptualization: NG, EAR, AB; Data curation: NG, EAR; Formal analysis: NG, EAR; Methodology: NG, EAR, AB; Project administration: NG; Visualization: NG; Writing – original draft: NG, EAR, AB, US; Writing – review & editing: NG, EAR, BJ, AB, US
