# Supplementary material for: Evaluations of digital public health interventions in the WHO Southeast Asia Region: a systematic literature review
Source: Int J Technol Assess Health Care. 2024 Dec 18;40(1):e78. doi: 10.1017/S026646232400045X (PMC11703627; doi:10.1017/S026646232400045X)
Supplement: Gudi et al. supplementary material [file S026646232400045Xsup001.docx]

**Supplementary Material**

**Table S1: Search strategy utilized in databases and corresponding article hits.**

| **S.no** | **Database** | **Filter** | **Hits** | **Date and Time (IST)** |
| --- | --- | --- | --- | --- |
| 1 | PubMed (NCBI)  (((Digital Health OR digital health technologies or Digital health interventions OR digital public health interventions OR Cell Phone OR smartphone OR Text Messaging OR mobile health OR mobile care OR mHealth OR ehealth OR electronic health OR Mobile applications OR TXT OR PXT OR MMS OR SMS OR short messaging service OR mobile communication OR mobile telecommunication OR telemedicine OR mobile technology OR cellular technology OR computer tablet OR pc tablet OR palmtop computer OR palm top computer OR pda computer OR pocket pc OR PDA phone OR blackberry OR palm pilot OR pilot palm OR wearables OR point of care devices OR Interactive voice response OR IVR OR GPRS OR Bluetooth OR GPS OR global positioning system OR Artificial intelligence OR Ai OR AI OR Machine learning OR ML OR Ml OR chat bots OR chat bot OR wearable*) AND (HTA OR health technology assessment OR biomedical technology assessment OR technology assessment OR technological assessment OR technological evaluation OR economic assessment OR economic evaluation OR cost-benefit analysis OR Cost-utility analysis OR cost-minimization analysis OR cost-effectiveness analysis OR Cost utility analysis OR cost minimization analysis OR cost effectiveness analysis OR CBA OR CUA OR CMA OR CEA)) AND (((WHO SEARO OR South East Asia Region OR SEAR OR WHO SEAR) OR (((Bangladesh OR India OR Bhutan OR Indonesia OR Maldives OR Myanmar OR Nepal OR Sri Lanka OR Thailand OR Timor-Leste)) OR (DPRK OR Democratic Republic of Korea OR North Korea OR Democratic People’s Republic of Korea))) | Human, English | 3739 | 01/01/2023  Time: 10.20 am |
| 2 | EMBASE (Elsevier)  'digital health' OR 'digital health technologies' OR 'digital health interventions' OR 'digital public health interventions' OR 'cell phone' OR smartphone OR 'text messaging' OR 'mobile health' OR 'mobile care' OR mhealth OR ehealth OR 'electronic health' OR 'mobile applications' OR txt OR pxt OR mms OR sms OR 'short messaging service' OR 'mobile communication' OR 'mobile telecommunication' OR telemedicine OR 'mobile technology' OR 'cellular technology' OR 'computer tablet' OR 'pc tablet' OR 'palmtop computer' OR 'palm top computer' OR 'pda computer' OR 'pocket pc' OR 'pda phone' OR blackberry OR 'palm pilot' OR 'pilot palm' OR wearables OR 'point of care devices' OR 'interactive voice response' OR ivr OR gprs OR bluetooth OR gps OR 'global positioning system' OR 'artificial intelligence' OR ai OR 'machine learning' OR ml OR 'chat bots' OR 'chat bot' OR wearable*:ab,ti AND hta OR 'health technology assessment' OR 'biomedical technology assessment' OR 'technology assessment' OR 'technological assessment' OR 'technological evaluation' OR 'economic assessment' OR 'economic evaluation' OR 'cost-benefit analysis' OR 'cost-utility analysis' OR 'cost-minimization analysis' OR 'cost-effectiveness analysis' OR 'cost utility analysis' OR 'cost minimization analysis' OR 'cost effectiveness analysis' OR cba OR cua OR cma OR cea:ti,ab AND 'who searo' OR 'south east asia region' OR sear OR 'who sear' OR bangladesh OR india OR bhutan OR indonesia OR maldives OR myanmar OR nepal OR 'sri lanka' OR thailand OR 'timor leste' OR dprk OR 'democratic republic of korea' OR 'north korea' OR 'democratic peoples republic of korea':ti,ab | Human, English | 926 | 01/01/2023  Time: 10.30 am |
| 3 | Web of Science (Clarivate)  **(AB=("Digital Health" OR "digital health technologies" OR "Digital health interventions" OR "digital public health interventions" OR "Cell Phone" OR smartphone OR "Text Messaging" OR "mobile health" OR "mobile care" OR mHealth OR ehealth OR "electronic health" OR "Mobile applications" OR TXT OR PXT OR MMS OR SMS OR "short messaging service" OR "mobile communication" OR "mobile telecommunication" OR telemedicine OR "mobile technology" OR "cellular technology" OR "computer tablet" OR "pc tablet" OR "palmtop computer" OR "palm top computer" OR "pda computer" OR "pocket pc" OR "PDA phone" OR blackberry OR "palm pilot" OR "pilot palm" OR wearables OR "point of care devices" OR "Interactive voice response" OR IVR OR GPRS OR Bluetooth OR GPS OR "global positioning system" OR "Artificial intelligence" OR Ai OR AI OR "Machine learning" OR ML OR Ml OR "chat bots" OR "chat bot" OR wearable*)) OR TI=("Digital Health" OR "digital health technologies" OR "Digital health interventions" OR "digital public health interventions" OR "Cell Phone" OR smartphone OR "Text Messaging" OR "mobile health" OR "mobile care" OR mHealth OR ehealth OR "electronic health" OR "Mobile applications" OR TXT OR PXT OR MMS OR SMS OR "short messaging service" OR "mobile communication" OR "mobile telecommunication" OR telemedicine OR "mobile technology" OR "cellular technology" OR "computer tablet" OR "pc tablet" OR "palmtop computer" OR "palm top computer" OR "pda computer" OR "pocket pc" OR "PDA phone" OR blackberry OR "palm pilot" OR "pilot palm" OR wearables OR "point of care devices" OR "Interactive voice response" OR IVR OR GPRS OR Bluetooth OR GPS OR "global positioning system" OR "Artificial intelligence" OR Ai OR AI OR "Machine learning" OR ML OR Ml OR "chat bots" OR "chat bot" OR wearable*) AND (TI=(HTA OR "health technology assessment" OR "biomedical technology assessment" OR "technology assessment" OR "technological assessment" OR "technological evaluation" OR "economic assessment" OR "economic evaluation" OR "cost-benefit analysis" OR "Cost-utility analysis" OR "cost-minimization analysis" OR "cost-effectiveness analysis" OR "Cost utility analysis" OR "cost minimization analysis" OR "cost effectiveness analysis" OR CBA OR CUA OR CMA OR CEA)) OR AB=(HTA OR "health technology assessment" OR "biomedical technology assessment" OR "technology assessment" OR "technological assessment" OR "technological evaluation" OR "economic assessment" OR "economic evaluation" OR "cost-benefit analysis" OR "Cost-utility analysis" OR "cost-minimization analysis" OR "cost-effectiveness analysis" OR "Cost utility analysis" OR "cost minimization analysis" OR "cost effectiveness analysis" OR CBA OR CUA OR CMA OR CEA) AND (TI=("WHO SEARO" OR "South East Asia Region" OR SEAR OR "WHO SEAR" OR Bangladesh OR India OR Bhutan OR Indonesia OR Maldives OR Myanmar OR Nepal OR "Sri Lanka" OR Thailand OR Timor-Leste OR DPRK OR "Democratic Republic of Korea" OR "North Korea" OR "Democratic People’s Republic of Korea")) OR AB=("WHO SEARO" OR "South East Asia Region" OR SEAR OR "WHO SEAR" OR Bangladesh OR India OR Bhutan OR Indonesia OR Maldives OR Myanmar OR Nepal OR "Sri Lanka" OR Thailand OR Timor-Leste OR DPRK OR "Democratic Republic of Korea" OR "North Korea" OR "Democratic People’s Republic of Korea")** | English | 40 | 01/01/2023  Time: 10.40 am |
| 4 | Scopus (Elsevier)  ( ( ( "Digital Health" OR "digital health technologies" OR "Digital health interventions" OR "digital public health interventions" OR "Cell Phone" OR smartphone OR "Text Messaging" OR "mobile health" OR "mobile care" OR mhealth OR ehealth OR "electronic health" OR "Mobile applications" OR txt OR pxt OR mms OR sms OR "short messaging service" OR "mobile communication" OR "mobile telecommunication" OR telemedicine OR "mobile technology" OR "cellular technology" OR "computer tablet" OR "pc tablet" OR "palmtop computer" OR "palm top computer" OR "pda computer" OR "pocket pc" OR "PDA phone" OR blackberry OR "palm pilot" OR "pilot palm" OR wearables OR "point of care devices" OR "Interactive voice response" OR ivr OR gprs OR bluetooth OR gps OR "global positioning system" OR "Artificial intelligence" OR ai OR ai OR "Machine learning" OR ml OR ml OR "chat bots" OR "chat bot" OR wearable* ) AND ( hta OR "health technology assessment" OR "biomedical technology assessment" OR "technology assessment" OR "technological assessment" OR "technological evaluation" OR "economic assessment" OR "economic evaluation" OR "cost-benefit analysis" OR "Cost-utility analysis" OR "cost-minimization analysis" OR "cost-effectiveness analysis" OR "Cost utility analysis" OR "cost minimization analysis" OR "cost effectiveness analysis" OR cba OR cua OR cma OR cea ) ) AND ( "community-based interventions" OR "community based interventions" OR "community interventions" OR "public interventions" OR "community health interventions" OR "public health interventions" OR "community based public health interventions" ) ) AND ( ( ( "WHO SEARO" OR "South East Asia Region" OR sear OR "WHO SEAR" ) OR ( ( ( bangladesh OR india OR bhutan OR indonesia OR maldives OR myanmar OR nepal OR "Sri Lanka" OR thailand OR timor-leste ) ) OR ( dprk OR "Democratic Republic of Korea" OR "North Korea" OR "Democratic People's Republic of Korea" ) ) ) ) | English | 498 | 01/01/2023  Time: 11.00 am |

**Table S2: List of articles excluded at full text stage along with reasons**

| **Sl no** | **Reference** | **Reason for Exclusion** |
| --- | --- | --- |
| 1 | Yadav S, Mishra S. Longitudinal Trial of a Smart Phone Application for Tele–Follow-Up of Thyroid Cancer Patients in Context of a Developing Country: Compliance, Satisfaction and Cost-Benefit Analysis. | Conference Abstract |
| 2 | Mehta S, Sharma S, Sharma S, Makkar JS, Yepes I, Torres M, Rodriguez D. TCT-395 Cost-effective, Innovative, Indigenous, Population-based and Telemedicine-guided, AMI Strategy for India’s most Populous State. Journal of the American College of Cardiology. 2017 Oct 31;70(18S):B162-. | Conference Abstract |
| 3 | Mehta S, Sharma S, Sharma S, Makkar JS, Yepes I, Torres M, Rodriguez D. TCT-395 Cost-effective, Innovative, Indigenous, Population-based and Telemedicine-guided, AMI Strategy for India’s most Populous State. Journal of the American College of Cardiology. 2017 Oct 31;70(18S):B162-. | Conference Abstract |
| 4 | Ananthakrishnan A, Shankar M, Chahar A, Kachroo K, Ameel M, Sharma J, Vsn M, Dang A. The Use of Mobile Health Technology in Promoting Infant Vaccine Adherence–A Health Technology Assessment. Value in Health. 2015 Nov 1;18(7):A559. | Conference Abstract |
| 5 | Jo Y, LeFevre AE, Ali H, Mehra S, Alland K, Shaikh S, Haque R, Pak ES, Chowdhury M, Labrique AB. mCARE, a digital health intervention package on pregnancy surveillance and care-seeking reminders from 2018 to 2027 in Bangladesh: a model-based cost-effectiveness analysis. BMJ open. 2021 Apr 1;11(4):e042553. | Duplicate |
| 6 | Kavitha S, Prasad NH, Samal CK, Hanumanthappa M. Evaluation of Cost benefit Analysis using One-R Supervised Machine Learning Algorithm for Healthcare.[Research Square] | Pre-print |
| 7 | Shah S, Singh K, Ali MK, Mohan V, Kadir MM, Unnikrishnan AG, Sahay RK, Varthakavi P, Dharmalingam M, Viswanathan V, Masood Q. Improving diabetes care: multi-component cardiovascular disease risk reduction strategies for people with diabetes in South Asia—the CARRS multi-center translation trial. Diabetes research and clinical practice. 2012 Nov 1;98(2):285-94. | Protocol |
| 8 | Chawla S, Chawla A, Chawla R, Jaggi S, Singh D, Trehan S. Trained nurse–operated teleophthalmology screening approach as a cost-effective tool for diabetic retinopathy. International Journal of Diabetes in Developing Countries. 2022 Jan 4:1-4. | Setting |
| 9 | Schwendicke F, Mertens S, Cantu AG, Chaurasia A, Meyer-Lueckel H, Krois J. Cost-effectiveness of AI for caries detection: randomized trial. Journal of dentistry. 2022 Apr 1;119:104080. | Setting |
| 10 | Neogi SB, John D, Sharma J, Kar R, Kar SS, Bhattacharya M, Tiwari K, Saxena R. Cost-effectiveness of point-of-care devices for detection of anemia in community settings in India. Clinical Epidemiology and Global Health. 2022 Mar 1;14. | Setting |
| 11 | Islam SM, Peiffer R, Chow CK, Maddison R, Lechner A, Holle R, Niessen L, Laxy M. Cost-effectiveness of a mobile-phone text messaging intervention on type 2 diabetes—A randomized-controlled trial. Health Policy and Technology. 2020 Mar 1;9(1):79-85. | Setting |
| 12 | Datta B, Prakash AK, Ford D, Tanwar PK, Goyal P, Chatterjee P, Vipin S, Jaiswal A, Trehan N, Ayyagiri K. Comparison of clinical and cost-effectiveness of two strategies using mobile digital x-ray to detect pulmonary tuberculosis in rural India. BMC Public Health. 2019 Dec;19:1-8. | Setting |
| 13 | Ferraris KP, Golidtum JP, Zuñiga BK, Bautista MC, Alcazaren JC, Seng K, Navarro JE. Recapitulating the Bayesian framework for neurosurgical outpatient care and a cost-benefit analysis of telemedicine for socioeconomically disadvantaged patients in the Philippines during the pandemic. Neurosurgical focus. 2020 Dec 1;49(6):E14. | Setting |
| 14 | Chandrakanth P, Chavan S, Verghese S, Gosalia H, Raman GV, Shettigar CK, Narendran V. Smartphone gonioscopy with a magnifying intraocular lens: A cost-effective angle imaging device. Journal of Glaucoma. 2022 May 1;31(5):356-60. | Study design |
| 15 | Subramanian S, Jose R, Lal A, Augustine P, Jones M, Gopal BK, Swayamvaran SK, Saroji V, Samadarsi R, Sankaranarayanan R. Acceptability, utility, and cost of a mobile health cancer screening education application for training primary care physicians in India. The Oncologist. 2021 Dec;26(12):e2192-9. | Study design |
| 16 | Jain A, Sahu R, Jain A, Gaumnitz T, Sethi P, Lodha R. Development and validation of a low-cost electronic stethoscope: DIY digital stethoscope. BMJ Innovations. 2021 Jun 30:bmjinnov-2021. | Study design |
| 17 | Jamthikar AD, Gupta D, Johri AM, Mantella LE, Saba L, Kolluri R, Sharma AM, Viswanathan V, Nicolaides A, Suri JS. Low-cost office-based cardiovascular risk stratification using machine learning and focused carotid ultrasound in an Asian-Indian cohort. Journal of Medical Systems. 2020 Dec;44:1-5. | Study design |
| 18 | Rout SK, Gabhale YR, Dutta A, Balakrishnan S, Lala MM, Setia MS, Bhuyan K, Manglani MV. Can telemedicine initiative be an effective intervention strategy for improving treatment compliance for pediatric HIV patients: Evidences on costs and improvement in treatment compliance from Maharashtra, India. PLoS One. 2019 Oct 8;14(10):e0223303. | Study design |
| 19 | Prinja S, Gupta A, Bahuguna P, Nimesh R. Cost analysis of implementing mHealth intervention for maternal, newborn & child health care through community health workers: assessment of ReMIND program in Uttar Pradesh, India. BMC Pregnancy and Childbirth. 2018 Dec;18:1-6. | Study design |
| 20 | Ramkumar V, John KR, Selvakumar K, Vanaja CS, Nagarajan R, Hall JW. Cost and outcome of a community-based paediatric hearing screening programme in rural India with application of tele-audiology for follow-up diagnostic hearing assessment. International Journal of Audiology. 2018 Jun 3;57(6):407-14. | Study design |
| 21 | Kaur G, Chauhan AS, Prinja S, Teerawattananon Y, Muniyandi M, Rastogi A, Jyani G, Nagarajan K, Lakshmi PV, Gupta A, Selvam JM. Cost-effectiveness of population-based screening for diabetes and hypertension in India: an economic modelling study. The Lancet Public Health. 2022 Jan 1;7(1):e65-73. | Study design |
| 22 | Rodrigues R, Bogg L, Shet A, Kumar DS, De Costa A. Mobile phones to support adherence to antiretroviral therapy: what would it cost the Indian National AIDS Control Programme?. Journal of the International AIDS Society. 2014 Jan;17(1):19036. | Study design |
| 23 | Singh M, Agarwal A, Sinha V, Kumar RM, Jaiswal N, Jindal I, Pant P, Kumar M. Application of handheld tele-ECG for health care delivery in rural India. International Journal of Telemedicine and Applications. 2014 Jan 1;2014:12-. | Study design |
| 24 | Sorwar G, Rahamn MM, Uddin R, Hoque MR. Cost and time effectiveness analysis of a telemedicine service in Bangladesh. Stud Health Technol Inform. 2016 Nov 24;231:127-34. | Study design |
| 25 | Yadav SK, Jha CK, Mishra SK, Mishra A. Smartphone-based application for tele-follow-up of patients with endocrine disorders in context of a LMIC: a compliance, satisfaction, clinical safety and outcome assessment. World Journal of Surgery. 2020 Feb;44:612-6. | Study design |
| 26 | Prinja S, Gupta A, Bahuguna P, Nimesh R. Cost analysis of implementing mHealth intervention for maternal, newborn & child health care through community health workers: assessment of ReMIND program in Uttar Pradesh, India. BMC Pregnancy and Childbirth. 2018 Dec;18:1-6. | Study design |
| 27 | Imtiaz SA, Krishnaiah S, Yadav SK, Bharath B, Ramani RV. Benefits of an android based tablet application in primary screening for eye diseases in a rural population, India. Journal of medical systems. 2017 Apr;41(4):49. | Study design |
| 28 | Peiris D, Praveen D, Mogulluru K, Ameer MA, Raghu A, Li Q, Heritier S, MacMahon S, Prabhakaran D, Clifford GD, Joshi R. SMARThealth India: a stepped-wedge, cluster randomised controlled trial of a community health worker managed mobile health intervention for people assessed at high cardiovascular disease risk in rural India. PLoS One. 2019 Mar 26;14(3):e0213708. | Study design |
| 29 | Tangka FK, Subramanian S, Edwards P, Cole-Beebe M, Parkin DM, Bray F, Joseph R, Mery L, Saraiya M. Resource requirements for cancer registration in areas with limited resources: analysis of cost data from four low-and middle-income countries. Cancer epidemiology. 2016 Dec 1;45:S50-8. | Study design |
| 30 | Aiga H, Huy TK, Nguyen VD. Cost savings through implementation of an integrated home-based record: a case study in Vietnam. Public Health. 2018 Mar 1;156:124-31. | Study design |

**Table S3: Study characteristics of included studies**

| ***Sl no*** | ***Study ID (Author, Year)*** | ***Title*** | ***Objective*** | ***Study Design*** | ***Country of Implementation*** | ***Disease / Condition/Procedure*** | ***Type of intervention*** | ***Type of economic evaluation*** | ***Perspective of Economic Evaluation*** |
| --- | --- | --- | --- | --- | --- | --- | --- | --- | --- |
| 1 | Menon 2021 | Health technology assessment of telemedicine applications in Northern borders of India | 1. To obtain the comparative cost estimates in providing these interventions by studying the costs involved in delivering telemedicine services and air transportation of casualties. 2. A qualitative assessment of the telemedicine implementation scenario in the peripheral hospitals and selected referral hospitals to identify the strengths, weaknesses, opportunities, and threats (SWOT) of telemedicine services in forward areas. | Multi-methods | India | Not Mentioned (to manage casualties among the armed forces at the Ladakh border) | Telemedicine | Cost-Effectiveness | Health system perspective |
| 2 | Angell 2021 | Cost-effectiveness of a Mobile technology-enabled primary care intervention for cardiovascular disease risk management in Rural Indonesia | a modeled cost-effectiveness analysis of the SMARThealth Extend intervention relative to usual care in the rural Indonesian province of Malang using a specifically developed Markov model. | Quasi-experimental study | Indonesia | cardiovascular disease risk management | m-Health | Cost-Effectiveness | Health system perspective |
| 3 | Jo 2021 | mCARE, a digital health intervention package on pregnancy surveillance and care-seeking reminders from 2018 to 2027 in Bangladesh: a model-based cost-effectiveness analysis | the objective of this study is to assess the cost-effectiveness of the mCARE programme, compared with the current paper-based status quo, over a 10-year analytic time horizon (2018–2027) to help guide investment decisions and to promote large-scale use and sustainability of mHealth intervention. | Quasi-experimental study | Bangladesh | Maternal and newborn health (MNH) services | m-health | Cost-Effectiveness | Societal perspective |
| 4 | Jo 2019 | Costs and cost-effectiveness analyses of mCARE strategies for promoting care seeking of maternal and newborn health services in rural Bangladesh | To examine the incremental cost-effectiveness between two mHealth programs, implemented from 2011 to2015 in rural Bangladesh:(1) Comprehensive mCARE package as an intervention group and (2) Basic mCARE package as a control group. | Quasi-experimental study | Bangladesh | Maternal and newborn health (MNH) services | m-health | Cost-Effectiveness | Program perspective (Societal) |
| 5 | Wongwai 2015 | A store-and-forward telemedicine for retinopathy of prematurity screen: is it cost-effective in Thailand? | To assess the value-for-money of telemedicine in screening for ROP in high-risk infants. | Prospective non-randomized open labelled Clinical trial | Thailand | Retinopathy of prematurity | Telemedicine | Cost-utility | Health Care Provider and Societal perspective |
| 6 | Salvadori 2020 | Appointment reminders to increase uptake of HIV retesting by at-risk individuals: a randomized controlled study in Thailand | A randomized controlled trial was conducted in Thailand to evaluate whether reminders could increase the uptake of HIV retesting by at-risk individuals. | 3 arm open labelled randomised controlled trial | Thailand | HIV | SMS remainders (mHealth) | Cost-Effectiveness | Societal perspective |
| 7 | Xie 2020 | Artificial intelligence for teleophthalmology-based diabetic retinopathy screening in a national programme: an economic analysis modelling study. | A cost-minimisation analysis was conducted to evaluate the potential savings of two deep learning approaches as compared with the current human assessment: a semiautomated deep learning model as a triage filter before secondary human assessment; and a fully automated deep learning model without human assessment. | Diagnostic Test Accuracy (DTA) | Singapore | diabetic retinopathy screening | Ai | Cost-minimization Analysis | Health System Perspective |
| 8 | Thakar 2018 | Comparison of telemedicine with in-person care for follow-up after elective neurosurgery: results of a cost-effectiveness analysis of 1200 patients using patient-perceived utility scores. | The authors evaluated the cost-effectiveness of TM consultations for follow-up care of a large population of patients who underwent neurosurgical procedures. | Retrospective observational study | India | Elective neurosurgery | Telemedicine | Cost-Effectiveness | Societal perspective |
| 9 | Arora 2017 | Cost-effectiveness analysis of telephone-based support for the management of pressure ulcers in people with spinal cord injury in India and Bangladesh. | To determine from a societal perspective the cost-effectiveness and cost-utility of telephone-based support for management of pressure ulcers | Multicentre, prospective, assessor blinded, parallel, pragmatic, randomised controlled trial. | India and Bangladesh | Pressure ulcers in people with spinal cord injury | telephone-based support | Cost-effectiveness analysis | Societal perspective |
| 10 | Nguyen 2016 | Cost-effectiveness of a National Telemedicine Diabetic Retinopathy Screening Program in Singapore. | To determine the incremental cost-effectiveness of a new telemedicine technician-based assessment relative to an existing model of family physician (FP) based assessment of diabetic retinopathy (DR) in Singapore from the health system and societal perspectives | Cohort (hypothetical cohort) | Singapore | Diabetic retinopathy | Telemedicine | Model-based, cost-effectiveness analysis | Societal perspective and Health Systems Perspective |
| 11 | Anchala 2015 | Evaluation of Effectiveness and Cost-Effectiveness of a Clinical Decision Support System in Managing Hypertension in Resource Constrained Primary Health Care Settings: Results from a Cluster Randomized Trial | To test the effectiveness and cost-effectiveness of Decision Support Systems (DSS) for BP management among Indian hypertensive patients. | Cluster Randomized Trial | India | Hypertension | Decision Support System | Cost-effectiveness analysis | Societal perspective |
| 12 | Modi 2020 | Costing and Cost-Effectiveness of a Mobile Health Intervention (ImTeCHO) in Improving Infant Mortality in TribalAreas of Gujarat, India: Cluster Randomized Controlled Trial | The study assessed the incremental cost per life-year saved as a result of the ImTeCHO intervention as compared to routine MNCH programs | A two-arm, parallel, stratified cluster randomized trial | India | Child Healthcare | m-health | Cost-effectiveness analysis | Program (Societal) and provider perspective |
| 13 | Rachapelle 2013 | The cost-utility of telemedicine to screen for diabetic retinopathy in India | To assess the cost-effectiveness of a telemedicine diabetic retinopathy (DR) screening program in rural Southern India that conducts 1-off screening camps (i.e., screening offered once) in villages and to assess the incremental cost-effectiveness ratios of different screening intervals. | A cost– utility analysis using a Markov model | India | Screening for Diabetic Retinopathy | Teleopthalmology | Cost– utility analysis | Healthcare provider and societal perspective |

Definitions of the various modalities:

1. **Telemedicine:** Menon 2021 defines it as **“**the use of telecommunication and information technology to enhance access to healthcare services in areas where medical infrastructure and technical expertise would not be consistently available”.(1)

2. **m health: WHO defines it as** “the use of mobile and wireless technologies to support the achievement of health objectives**.”** (2)

3. **Telephone-based support:** from the study by Arora et al. (2017), we infer that it is the one-to-one support provided over a call for the management of pressure ulcers. (3)

4**. Decision support system (DSS):** Anchala et al. 2015 define DSS as “tools that help clinicians decide on a course of action in response to an understanding of the patient's status.”(4)

5. **Artificial Intelligence** is defined as “the simulation of human intelligence processes by machines, especially computer systems.”(5)

**References**

1. Menon AK, Adhya S, Kanitkar M. Health technology assessment of telemedicine applications in Northern borders of India. *Medical Journal Armed Forces India*. 2021;77(4):452-458. doi:10.1016/j.mjafi.2021.03.007

2. mHealth: New horizons for health through mobile technologie. WHO | Regional Office for Africa. Published January 12, 2024. Accessed January 13, 2024. https://www.afro.who.int/publications/mhealth-new-horizons-health-through-mobile-technologie

3. Cost-effectiveness analysis of telephone-based support for the management of pressure ulcers in people with spinal cord injury in India and Bangladesh | Spinal Cord. Accessed June 22, 2023. https://www.nature.com/articles/sc201787

4. Evaluation of Effectiveness and Cost‐Effectiveness of a Clinical Decision Support System in Managing Hypertension in Resource Constrained Primary Health Care Settings: Results From a Cluster Randomized Trial | Journal of the American Heart Association. Accessed June 22, 2023. https://www.ahajournals.org/doi/full/10.1161/JAHA.114.001213

5. What is Artificial Intelligence and How Does AI Work? | Definition from TechTarget. Enterprise AI. Accessed January 13, 2024. https://www.techtarget.com/searchenterpriseai/definition/AI-Artificial-Intelligence

**Table S4: Results and limitations of the included studies**

| ***Sl no*** | ***Study ID (Author, Year)*** | ***Title*** | **Results** | **Study limitations (as reported)** | **Funding and Role of funder** |
| --- | --- | --- | --- | --- | --- |
| **1** | Menon 2021 | Health technology assessment of telemedicine applications in Northern borders of India | 1. One-third of the casualties could be stabilized using telemedicine consultations. 2. No fatality was reported in the 269 cases managed by Telemedicine. | 1. The cost calculation did not include overhead costs (hospital building, electricity, and telephone) and salaries of the telemedicine unit's employees.  2. The estimation of air transportation costs did not consider the costs of drugs and consumables. This has led to conservative cost estimates in the present study. | Not Reported |
| **2** | Angell 2021 | Cost-effectiveness of a Mobile technology-enabled primary care intervention for cardiovascular disease risk management in Rural Indonesia | 1. On average, annual treatment costs for CVD management were $83 under current care and $144 for those receiving the intervention. (additional primary care visits were included in the intervention) 2. There were 16,407 fewer CVD events and 7,113 fewer CVD-related deaths for the group receiving the intervention over 10 years. 3. The intervention was found to yield an ICER of $4288 per DALY averted and $3681 per major CVD event avoided relative to usual care. | 1. The data for the model parameters were developed using data collected from 8 villages, hence generalization was challenging.  2. The analysis did not include lost income as a result of experiencing a major CVD event or caring for someone who has. | This study was funded by a grant from Give2Asia on the recommendation of  Pfizer Foundation and Australian National Health and Medical Research (NHMRC) program [APP1052555]; NHMRC principal research fellowship [APP1136898 to A. Patel]; an NHMRC Early Career Fellowship  [APP1141392 to T.L.]; an NHMRC Early Career Fellowship [APP1161527 to A. Palagyi]. S.J. is the recipient of an NHMRC Principal Research Fellowship [1119443] .  **Role of the funder:** The funding sources had no role in the design and conduct of the study; collection, management, analysis and interpretation of the data; preparation, review or approval of the manuscript; nor the decision to submit the manuscript for publication. |
| **3** | Jo 2021 | mCARE, a digital health intervention package on pregnancy surveillance and care-seeking reminders from 2018 to 2027 in Bangladesh: a model-based cost-effectiveness analysis | 1. The mCARE program to avert 3076 deaths by 2027 at an incremental cost of $43 million relative to the status quo, which is translated to $462 per DALY averted.  2. The societal costs were estimated to be $115 million for mCARE programme (48% of which are programme costs, 35% user costs and 17% provider costs). 3. With the continued implementation and geographical scaling-up, the mCARE programme improved its cost-effectiveness from $1152 to $462 per DALY averted from 5 to 10 years. | 1. The cost-effectiveness was draw with estimates from observed data and a range of assumptions regarding the population and service coverage metrics using LiST to forecast health impact and costs.  2. The model does not incorporate detailed complexities between preventive and curative care. | UBS Optimus Foundation (Grant No.  11053414).  **Role of the funder:** Not reported |
| **4** | Jo 2019 | Costs and cost-effectiveness analyses of mCARE strategies for promoting care seeking of maternal and newborn health services in rural Bangladesh | 1. The addition of SMS and home visit reminders based on a mobile phone-facilitated pregnancy surveillance system was highly cost effective at a cost per DALY averted of $31 (95% uncertainty range: $19–81). 2. The comprehensive mCARE program had at least 88% probability of being highly cost-effective as compared to the basic mCARE program based on the threshold of Bangladesh’s GDP per capita. | 1. The mCARE I study was a pilot study on a quasi-experimental design, which did not provide enough statistical power and adjustment for confounding factors in evaluating mortality impact.  2. The cost adjustment for standardized estimations to a population of 1 million may not systematically incorporate potential changes with scaling up. | UBS Optimus Foundation  **Role of the funder:** Not reported |
| **5** | Wongwai 2015 | A store-and-forward telemedicine for retinopathy of prematurity screen: is it cost-effective in Thailand? | 1. The total capital cost for telemedicine to the health provider was 951,000 THB per year. 2. With the base case analysis of 400 children screened per year per RetCam, the performance of screening and diagnosis of Retinopathy of Prematurity (ROP) using telemedicine (100% sensitivity and 97.8% specificity) was higher compared with the current method (88.9% sensitivity and 93.4% specificity). 3. The incremental cost to society of telemedicine compared with the current practice was 837 THB. | 1. The utility value used in the analysis is not the utility of Thai children who have visual impairment. 2. The wide sensitivity range of the RetCam in detecting ROP-RT, may have resulted from the small sample size of the clinical trial. | This study was funded by Siriraj Hospital, Faculty of Medicine, Mahidol University. The Health Intervention and Technology Assessment Program (HITAP) is funded by the Thailand Research Fund under the Senior Research Scholar on Health Technology Assessment (RTA5580010), the National  Health Security Office, the Health System Research Institute and the Bureau of Health Policy and Strategy, Ministry of Public Health.  **Role of the funder:** Not reported |
| **6** | Salvadori 2020 | Appointment reminders to increase uptake of HIV retesting by at-risk individuals: a randomized controlled study in Thailand | 1. The proportion presenting for HIV retesting within seven months was 11.2% (24/215) in the control arm, versus 19.3% (42/218) in “No Appointment but Reminder” (p = 0.023) and 36.7% (80/218) in “Appointment & Reminder” (p < 0.001).  2. The incremental cost-effectiveness ratios of “No Appointment but Reminder” and “Appointment & Reminder” compared to the control arm were respectively USD 0.05 and USD 0.14 per participant for each 5% increase in HIV retesting uptake within seven months. | 1. The researchers of this study were unable to know if the clients presented for testing in other centers.  2. The selection of study participants was based on a risk assessment performed by counselors, which could vary across counselors | The Napneung project was supported by a grant from Expertise France through the 5% Initiative program (14SANIN204).  **Role of the funder:** Not reported |
| **7** | Xie 2020 | Artificial intelligence for teleophthalmology-based diabetic retinopathy screening in a national programme: an economic analysis modelling study. | 1. Amongst the Fully automated, semi-automated models and the human assessment methods, the semi-automated screening model was the least expensive (US$62 per patient per year).  2. The savings to the Singapore health system associated with switching to the semi-automated model are estimated to be $489 000, which is roughly 20% of the current annual screening cost. | 1. Generalizability of research findings is challenging, however, the model could be adapted. 2. Diabetic retinopathy diagnoses was considered and diabetic macular oedema cases with mild referable diabetic retinopathy were not considered which might under-estimate the cost saving from the fully automated model. | This project received funding from a National Medical Research Council Health Service Research Grant, Ministry of Health. The diabetes study in nephropathy and other microvascular complications received funding from a National Medical Research Council Large Collaborative Grant.  **Role of the funder:** The National Medical Research Council Health Service, Ministry of Health, Singapore had no role in the design and conduct of the study; collection, management, analysis, and interpretation of the data; preparation, review, or approval of the Article; and decision to submit the Article for publication. |
| **8** | Thakar 2018 | Comparison of telemedicine with in-person care for follow-up after elective neurosurgery: results of a cost-effectiveness analysis of 1200 patients using patient-perceived utility scores. | 1. TM was found to be more cost-effective (Indian rupee [INR] 2630 per patient) compared to routine care (INR 6848 per patient). 2. TM was nore effective and less expensive than routine care ((ICER value of -39,400 INR/unit of effectiveness). 3. Sensitivity analysis revealed that cost-effectiveness of TM was most sensitive to changes in the number of TM patients, utility and success rate of TM, and travel distance to the TM center | 1. The heterogeneous clinical profile of the patients in the two groups (TM vs Routine care) could have been a source of selection bias. | Not reported |
| **9** | Arora 2017 | Cost-effectiveness analysis of telephone-based support for the management of pressure ulcers in people with spinal cord injury in India and Bangladesh. | 1. The mean (95% confidence interval) between-group difference for the reduction in size of pressure ulcers was 0.53 (−3.12 to 4.32) cm2, favoring the intervention group. 2. The corresponding QALYs were 0.027 (0.004–0.051), favoring the intervention group.  3. The mean total cost per participant in the intervention group was INR 43 781 (USD 2460) compared to INR 42 561 (USD 2391) for the control group.  4. The per-participant cost of delivering the intervention was INR 2110 (USD 119). The incremental cost-effectiveness ratio was INR 2306 (USD 130) per additional cm2 reduction in the size of the pressure ulcer and INR 44 915 (USD 2523) per QALY gained. | 1. The productivity losses associated with prolonged bed rest were not incorporated. 2. The long-term cost-effectiveness of the intervention was not estimated as the outcomes were measured at 12 weeks. | Not reported |
| **10** | Nguyen 2016 | Cost-effectiveness of a National Telemedicine Diabetic Retinopathy Screening Program in Singapore. | 1. The telemedicine-based DR screening model had significantly lower costs (total cost savings of S$173 per person) while generating similar QALYs compared with the physician-based model (i.e., 13.1 QALYs). 2. The cost savings are S$144 per person (from health systems perspective). | 1. The study did not account for the benefits associated with freeing FPs' time from grading DR images for other tasks.  2. The transition probabilities across the health states did not vary with age because of a lack of data in the literature. 3. The study did not account for the cost of time spent waiting for the result incurred by SiDRP patients. | The authors received funding from the Singapore Ministry of Health, Grant Reference AIC/RPDD/SIDRP/SERI/FY2013/0018 and AIC/HPD/FY2016/0912.  **Role of the funder:** Not reported |
| **11** | Anchala 2015 | Evaluation of Effectiveness and Cost-Effectiveness of a Clinical Decision Support System in Managing Hypertension in Resource Constrained Primary Health Care Settings: Results from a Cluster Randomized Trial | 1. Statistically significant differences were found in the DSS arm when unadjusted mean SBP (139.9; 95% CI: 135.1 to 144.8) at the 12th month was compared to the unadjusted mean SBP (151.1; 95% CI: 146.9 to 155.3) at 0 months (P<0.001). 2. Statistically significant differences were found in DSS arm when unadjusted mean DBP (84.3; 95% CI: 82.0 to 86.5) at the 12th month was compared to the unadjusted mean DBP (89.7; 95% CI: 87.7 to 91.7) at 0 months (P<0.001).  3. The average costs per patient for the CBS group patient ($344.69) divided by the average reduction in SBP over a 12‐month period (3.59 mm Hg) yielded a CER of $96.01 per mm reduction in SBP for the CBS group; the corresponding CER for DSS intervention arm was $36.57 ($370.48 divided by 10.13) per mm reduction in SBP. | 1. The study did not have usual care group.  2. Generalizability of the findings to urban areas would be limited. | This work was supported by a Wellcome Trust Capacity Strengthening Strategic Award to the Public Health Foundation of India and a consortium of UK universities.  **Role of the funder:** The funders had no role in the study design and analysis. |
| **12** | Modi 2020 | Costing and Cost-Effectiveness of a Mobile Health Intervention (ImTeCHO) in Improving Infant Mortality in TribalAreas of Gujarat, India: Cluster Randomized Controlled Trial | 1. From the 5754 live births (3014 in the intervention arm, 2740 in the control arm) reported in the study area, per protocol analysis showed that the implementation of ImTeCHO resulted in saving 11 infant deaths per 1000 live births in the study area at an annual incremental cost of US $163,841, which is equivalent to US $54,360 per 1000 live births. 2. ImTeCHO is a cost-effective intervention from a program perspective at an incremental cost of US $74 per life-years saved or US $5057 per death averted. | 1. The study did not assess the health care input cost or time spent by health workers in training, supportive supervision by medical officers, and other supervisors from the health system. | This study was funded by the Indian Council of Medical Research, John D and Catherine T MacArthur Foundation, and the World Health Organization. We acknowledge support from Argusoft India Ltd in designing the ImTeCHO software and mobile app. We thank the project team from SEWA-Rural for their efforts toward implementation and evaluation. We thank the local community of the study area, ASHAs, primary health care staff, and district and state health officers for their support with the intervention.  **Role of the funder:** Not reported |
| **13** | Rachapelle 2013 | The cost-utility of telemedicine to screen for diabetic retinopathy in India | 1. The current rural teleophthalmology program was cost-effective ($1320 per QALY) compared with no screening from a health provider perspective.  2. Screening intervals of up to a frequency of screening every 2 years also were cost-effective, but annual screening was not (>$3183 per QALY).  3.From a societal perspective, telescreening up to a frequency of once every 5 years was cost-effective, but not more frequently. | 1. The model assumed 100% attendance of the initial cohort at each subsequent DR screening. 2. The study did not include the costs of establishing and maintaining an administrative system for regular (i.e., every 1 to 5 years) screening (e.g., setting up screening databases, contacting screening participants, monitoring attendance) because empirical data on these costs are not available.  3. The study did not include potential societal cost-savings of averting costs associated with blindness, such as productivity gains, and this may underestimate cost-effectiveness. | This study was funded by a grant from Sightsavers.  **Role of the funder:** The funding organization had no role in the design or conduct of this research. |

**Table S5: Assessment dimension from the core model of HTA**

| **Sl no** | **Study id** | ***Health problem and current use of technology (CUR)*** | ***Description and technical characteristics of technology (TEC)*** | ***Safety (SAF)*** | ***Clinical effectiveness (EFF)*** | ***Costs and economic evaluation (ECO)*** | ***Ethical analysis (ETH)*** | ***Organizational aspects (ORG)*** | ***Patients and Social aspects (SOC)*** | ***Legal aspects (LEG)*** |
| --- | --- | --- | --- | --- | --- | --- | --- | --- | --- | --- |
| **1** | Menon 2021 | Yes | Yes | Yes | Yes | Yes | Yes | Yes | Yes | Yes |
| **2** | Angell 2021 | Yes | Yes | No | Yes | Yes | No | Yes | No | No |
| **3** | Jo 2021 | Yes | Yes | No | Yes | Yes | No | Yes | Yes | No |
| **4** | Jo 2019 | Yes | Yes | No | Yes | Yes | No | Yes | No | No |
| **5** | Wongwai 2015 | Yes | Yes | No | Yes | Yes | No | Yes | No | No |
| **6** | Salvadori 2020 | Yes | Yes | No | Yes | Yes | No | Yes | Yes | No |
| **7** | Xie 2020 | Yes | Yes | No | Yes | Yes | No | Yes | Yes | No |
| **8** | Thakar 2018 | Yes | Yes | No | Yes | Yes | No | Yes | Yes | No |
| **9** | Arora 2017 | Yes | Yes | No | Yes | Yes | No | Yes | No | No |
| **10** | Nguyen 2016 | Yes | Yes | No | Yes | Yes | No | Yes | No | No |
| **11** | Anchala 2015 | Yes | Yes | No | Yes | Yes | No | Yes | No | No |
| **12** | Modi 2020 | Yes | Yes | Yes | Yes | Yes | Yes | Yes | Yes | No |
| **13** | Rachapelle 2013 | Yes | Yes | Yes | Yes | Yes | No | Yes | Yes | No |

**Figure S1:** Assessment of dimensions from the EUnetHTA Core Model 3.0 of HTA (1)

**Table S6:** Critical Appraisal using JBI Economic Evaluation Tool

| **Study id** | **1. Is there a well-defined question?** | **2. Is there comprehensive description of alternatives?** | **3. Are all important and relevant costs and outcomes for each alternative identified?** | **4. Has clinical effectiveness been established?** | **5. Are costs and outcomes measured accurately?** | **6. Are costs and outcomes valued credibly?** | **7. Are costs and outcomes adjusted for differential timing?** | **8. Is there an incremental analysis of costs and consequences?** | **9. Were sensitivity analyses conducted to investigate uncertainty in estimates of cost or consequences?** | **10. Do study results include all issues of concern to users?** | **11. Are the results generalizable to the setting of interest in the review?** | **Overall appraisal** | **Classification** |
| --- | --- | --- | --- | --- | --- | --- | --- | --- | --- | --- | --- | --- | --- |
| Menon 2021 | Yes | Yes | Yes | Yes | Yes | Yes | No | Yes | No | No | Yes | 72.7 | Poor quality |
| Angell 2021 | Yes | Yes | Yes | Yes | Yes | Yes | Yes | Yes | Yes | Yes | Yes | 100 | Well conducted |
| Jo 2021 | Yes | Yes | Yes | Yes | Yes | Yes | Yes | Yes | Yes | Yes | Yes | 100 | Well conducted |
| Jo 2019 | Yes | Yes | Yes | Yes | Yes | Yes | Yes | Yes | Yes | Unclear | Yes | 90.9 | Well conducted |
| Wongwai 2015 | Yes | Yes | Yes | Yes | Yes | Yes | Yes | Yes | Yes | No | Yes | 90 | Well conducted |
| Salvadori 2020 | Yes | Yes | Yes | Yes | Yes | Yes | Unclear | Yes | No | Unclear | Unclear | 63.6 | Poor quality |
| Xie 2020 | Yes | Yes | Yes | Yes | Yes | Yes | Unclear | Yes | Yes | No | Yes | 81.8 | Well conducted |
| Thakar 2018 | Yes | Yes | Yes | Yes | Yes | Yes | Yes | Yes | Yes | Yes | Yes | 100 | Well conducted |
| Arora 2017 | Yes | Yes | Yes | Yes | Yes | Yes | Yes | Yes | Yes | No | Yes | 90.9 | Well conducted |
| Nguyen 2016 | Yes | Yes | Yes | Yes | Yes | Yes | Yes | Yes | Yes | Yes | Yes | 100 | Well conducted |
| Anchala 2015 | Yes | Yes | Unclear | Yes | Yes | Yes | Yes | Yes | Yes | Yes | Yes | 90.9 | Well conducted |
| Modi 2020 | Yes | Yes | Yes | Yes | Yes | Yes | Yes | Yes | Yes | Yes | Yes | 100 | Well conducted |

**Figure S2:** Critical appraisal of included studies using the JBI Economic Evaluation Tool (2)

**Table S7:** Completeness of reporting

| **Study ID** | Menon 2021 | Angell 2021 | Jo 2021 | Jo 2019 | Wongwai 2015 | Salvadori 2020 | Xie 2020 | Thakar 2018 | Arora 2017 | Nguyen 2016 | Anchala 2015 | Modi 2020 | Rachapelle 2013 |
| --- | --- | --- | --- | --- | --- | --- | --- | --- | --- | --- | --- | --- | --- |
| 1. Identify the study as an economic evaluation and specify the interventions being compared. | R | R | R | R | R | NR | R | R | R | R | R | R | R |
| 2. Provide a structured summary that highlights context, key methods, results, and alternative analyses. | NR | R | R | R | R | R | R | R | R | R | R | R | R |
| 3. Give the context for the study, the study question, and its practical relevance for decision making in policy or practice. | R | R | R | R | R | R | R | NR | R | R | NR | R | R |
| 4. Indicate whether a health economic analysis plan was developed and where available. | R | R | R | R | R | R | R | R | R | R | R | R | R |
| 5. Describe characteristics of the study population (such as age range, demographics, socioeconomic, or clinical characteristics). | R | R | R | R | R | R | R | R | R | R | R | R | R |
| 6. Provide relevant contextual information that may influence findings. | R | R | R | R | R | R | R | R | R | R | R | R | R |
| 7. Describe the interventions or strategies being compared and why chosen. | R | R | R | R | R | R | R | R | R | R | R | R | R |
| 8. State the perspective(s) adopted by the study and why chosen. | R | R | R | R | R | R | R | R | R | R | R | R | R |
| 9. State the time horizon for the study and why appropriate | R | R | R | R | R | R | R | R | R | R | R | R | R |
| 10. Report the discount rate(s) and reason chosen. | NR | R | R | R | R | NR | R | R | R | R | R | R | R |
| 11. Describe what outcomes were used as the measure(s) of benefit(s) and harm(s). | R | R | R | R | R | R | NA | R | R | R | R | R | R |
| 12. Describe how outcomes used to capture benefit(s) and harm(s) were measured. | R | R | R | R | R | R | NA | R | R | R | R | R | R |
| 13. Describe the population and methods used to measure and value outcomes. | R | R | R | R | R | R | NA | R | R | R | R | R | R |
| 14. Describe how costs were valued. (Oppourtunity cost) | R | R | R | R | R | R | R | R | R | R | R | R | R |
| 15. Report the dates of the estimated resource quantities and unit costs, plus the currency and year of conversion. | NR | R | R | R | R | R | R | R | R | R | R | R | R |
| 16. If modelling is used, describe in detail and why used. Report if the model is publicly available and where it can be accessed. | NA | R | R | NA | R | NA | R | R | NA | R | NA | R | R |
| 17. Describe any methods for analysing or statistically transforming data, any extrapolation methods, and approaches for validating any model used. | NR | R | R | R | R | NR | R | R | R | R | R | R | R |
| 18. Describe any methods used for estimating how the results of the study vary for subgroups. | NR | R | R | R | R | NR | R | R | R | R | R | R | R |
| 19. Describe how impacts are distributed across different individuals or adjustments made to reflect priority populations. | NR | R | R | R | R | R | R | R | R | R | R | R | R |
| 20. Describe methods to characterise any sources of uncertainty in the analysis. | NR | R | R | R | R | NR | R | R | R | R | R | R | R |
| 21. Describe any approaches to engage patients or service recipients, the general public, communities, or stakeholders (such as clinicians or payers) in the design of the study. | NR | R | R | R | NR | NR | NR | NR | NR | R | R | R | R |
| 22. Report all analytic inputs (such as values, ranges, references) including uncertainty or distributional assumptions. | NA | R | R | NA | R | NA | R | R | NA | R | R | R | R |
| 23. Report the mean values for the main categories of costs and outcomes of interest and summarise them in the most appropriate overall measure. | NR | R | R | R | R | R | R | R | R | R | R | R | R |
| 24. Describe how uncertainty about analytic judgments, inputs, or projections affect findings. Report the effect of choice of discount rate and time horizon, if applicable. | NR | R | R | R | R | NR | R | R | R | R | R | R | R |
| 25. Report on any difference patient/service recipient, general public, community, or stakeholder involvement made to the approach or findings of the study | NR | R | R | R | NR | NR | NR | NR | NR | NR | R | R | R |
| 26. Study findings, limitations, generalisability, and current knowledge | R | R | R | R | R | R | R | R | R | R | R | R | R |
| 27. Describe how the study was funded and any role of the funder in the identification, design, conduct, and reporting of the analysis | NR | R | R | R | R | R | R | NR | NR | R | R | R | R |
| 28. Report authors conflicts of interest according to journal or International Committee of Medical Journal Editors requirements. | R | R | R | R | R | R | R | R | R | R | R | R | R |

**Legend**

| Reported | R |
| --- | --- |
| Not Reported | NR |
| Not Applicable | NA |

**Figure S3:** Colum chart representing the completeness of reporting according to the CHEERS 2022 checklist (3)

**References**

1. HTACoreModel3.0.pdf [Internet]. [cited 2023 Jun 22]. Available from: https://www.eunethta.eu/wp-content/uploads/2018/01/HTACoreModel3.0.pdf

2. Joanna Briggs Institute 2017. Critical Appraisal Checklist for Economic Evaluations. 2017.[Internet]. [cited 2023 Jun 22]. Available from: https://jbi.global/sites/default/files/2019-05/JBI_Critical_Appraisal-Checklist_for_Economic_Evaluations2017_0.pdf

3.Consolidated Health Economic Evaluation Reporting Standards (CHEERS) 2022 Explanation and Elaboration: A Report of the ISPOR CHEERS II Good Practices Task Force - ScienceDirect [Internet]. [cited 2023 Jun 22]. Available from: https://www.sciencedirect.com/science/article/pii/S1098301521017952
